# Host genotype and exercise exhibit species-level selection for members of the gut bacterial communities in the mouse digestive system

**DOI:** 10.1038/s41598-020-65740-4

**Published:** 2020-06-02

**Authors:** R. A. Dowden, L. R. McGuinness, P. J. Wisniewski, S. C. Campbell, J. J. Guers, M. Oydanich, S. F. Vatner, M. M. Häggblom, L. J. Kerkhof

**Affiliations:** 1 Department of Kinesiology and Health, Rutgers, the State University of New Jersey, New Brunswick, NJ 08901 USA; 2 Department of Marine and Coastal Sciences, Rutgers, the State University of New Jersey, New Brunswick, NJ 08901 USA; 3 Department of Biology, Behavioral Neuroscience, and Health Sciences, Rider University, Lawrenceville, NJ 08648 USA; 4 Department of Cell Biology and Molecular Medicine, Rutgers, the State University of New Jersey, Newark, NJ USA; 5 Department of Biochemistry and Microbiology, Rutgers, the State University of New Jersey, New Brunswick, NJ 08901 USA

**Keywords:** Bacterial host response, Microbiome

## Abstract

The mammalian gut microbiome can potentially impact host health and disease state. It is known that the mouse-genome, eating-behavior, and exercise-status promotes higher taxonomic rank-level alterations (e.g. family to phyla-level) of the gut microbiota. Here, host genotype or activity status was investigated to determine if selection of individual bacterial species or strains could be discerned within the murine digestive system. For this study, the fecal bacterial community of adenylyl cyclase 5 knock-out (AC5KO, n = 7) mice or their wild-type (WT, n = 10) littermates under exercise or sedentary conditions were profiled by sequencing rRNA operons. AC5KO mice were chosen since this genotype displays enhanced longevity/exercise capacity and protects against cardiovascular/metabolic disease. Profiling of rRNA operons using the Oxford MinION yielded 65,706 2-D sequences (after size selection of 3.7–5.7 kb) which were screened against an NCBI 16S rRNA gene database. These sequences were binned into 1,566 different best BLAST hits (BBHs) and counted for each mouse sample. Non-metric multidimensional scaling (NMDS) of the gut microbial community demonstrated clustering by physical activity (p = 0.001) but not by host genotype. Additionally, sequence similarity and phylogenetic analysis demonstrated that different bacterial species (closely related to *Muribaculum intestinale* and *Parasutterella excrementihominis*) inhabit AC5KO or WT mice depending on activity status. Other bacterial species of the gut microbiota did not follow such patterning (e.g. *Turicibacter sanguinis* and *Turicimonas muris*). Our results support the need of improved taxonomic resolution for better characterization of bacterial communities to deepen our understanding of the role of the gut microbiome on host health.

## Introduction

Accumulating evidence suggests that the gut microbiome contributes to the development of chronic diseases such as obesity^1,2^, type 2 diabetes^2–5^ inflammatory bowel disease^6^, and cancer^7,8^. Additionally, aging^9^, toxin/radiation exposure^10,11^, or exercise training^12,13^ are also associated with changes in the mouse gut microbiome. Furthermore, several groups have demonstrated the role of host genotype^14^, behavior (e.g. diet^15–17^, and activity level^12,18^) in modulating human gut microbial communities. Unfortunately, most of these prior studies have utilized short regions of the 16S rRNA gene to monitor changes in the gut microbiome which are often reported at the genus to phylum levels. This can obscure potential changes in the digestive system that may arise at the level of bacterial species or strains. Recent improvements in sequencing platforms, such as the Oxford Nanopore Technologies (ONT) MinION, provide much longer reads^19^ with the potential for higher resolution microbiome analysis^20,21^. For example, efforts to characterize microbial communities by sequencing near full-length ribosomal operons have shown robust species-level identification for model communities^22,23^ or complex natural samples^24^. Although the MinION has a higher error rate than Illumina, the read accuracy has improved in recent years to 92%^25^. Furthermore, it has been shown that near full-length 16S rRNA genes with ~80% identity can be correctly assigned to the original source 16S rRNA genes using Discontiguous MegaBLAST for accurate OTU calling^24^. Additionally, consensus building of MinION raw reads from the same OTU can greatly reduce errors and provide species-level resolution^24^.

For this study, we wanted to test if two mouse models were colonized by different bacterial species or strains within their digestive systems using a long-read approach. AC5KO mice were chosen due to their enhanced phenotype with an improved lifespan^26–29^, protection against obesity/type 2 diabetes^30^, improved antioxidant defenses^28,31^, and an enhanced exercise capacity^32,33^ when compared to age-matched WT littermates. The purpose of this study was to understand if the microbial community composition in the AC5KO mice digestive tract differed from their WT littermates and how this was affected by exercise training. Our hypothesis was that the AC5KO animals have a different gut microbiota compared to WT mice (potentially at the species level) under exercise or sedentary conditions.

Here, bacterial rRNA operons from AC5KO and WT mouse fecal samples were analyzed using the ONT MinION sequencing platform. Although there are not a large number of individual mice being analyzed here, this study represents a proof-of-concept for using rRNA operon profiling to characterize the bacterial gut community and obtain species- and strain-level resolution. Specifically, we show how to analyze rRNA operon sequences by similarity clustering to assess whether novel bacterial species exist in complex samples. Our data indicates that all mice harbor different *Muribaculum* species across treatments (i.e. WT male sedentary mice [n = 6]; WT male exercise mice [n = 4]; AC5KO male and 1 female exercise mice [n = 3]; and AC5KO male sedentary mice [n = 4]). While AC5KO exercise/sedentary and WT exercise mice contained a different *Parasutterella* spp. compared with WT sedentary mice. This observed species-level difference in gut microbiota profiles suggest that host factors (e.g. biological or behavioral) are selecting for specific groups of bacteria within the gut and may ultimately contribute to critical aspects of the AC5KO phenotype (e.g. improved longevity, increased glucose and insulin tolerance, enhanced exercise tolerance). Although future studies will utilize newer Nanopore chemistries and base calling algorithms for rRNA operons profiling, the data analysis approach presented here is applicable for discerning cryptic variations within members of the bacterial communities that cannot be ascertained by screening short, variable regions against a 16S rRNA gene database. This ability to discern species-level and strain-level differences between gut bacterial microbiota will greatly enhance our understanding of which microbes to target for further culturing/physiological studies; could lead to new clinical tools for optimizing the gut microbiome; and provides insight into how gut microbes and hosts interact to promote health.

## Results

### Ribosomal RNA operon sequencing

**Figure 1:**
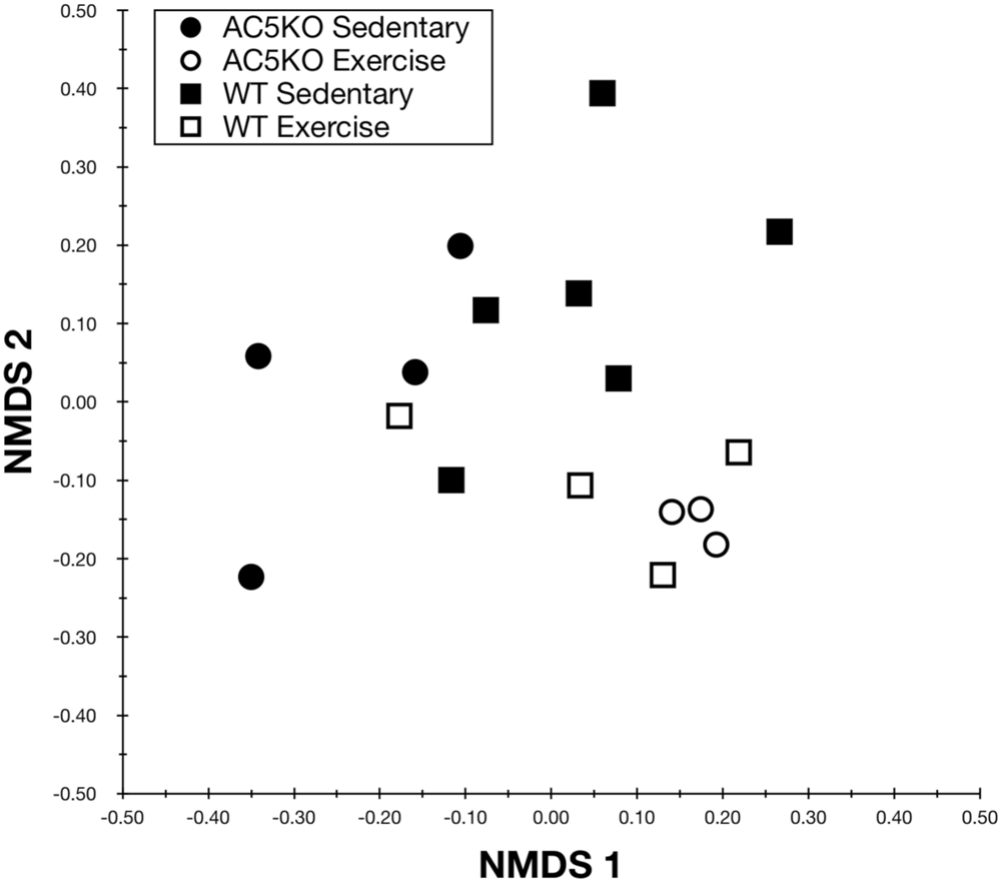
Non-metric multidimensional scaling analysis based on Bray Curtis similarity of the microbiome for the various genotypes (AC5KO or WT) and physical activity states (exercise or sedentary).

As with our prior work on MinION rRNA operon profiling^24^, we utilized the first Oxford 2D sequencing chemistry for this study (which is no longer commercially available). The 2D method provided increased read accuracy by sequencing both strands of the amplicon and generating a consensus with Albacore 2.1 basecalling software, albeit with lower raw read numbers. Two MinION sequencing libraries were created for rRNA operon amplicons from AC5KO and WT mice fecal samples and run for 18 or 22 h. A total of 382,731 total raw reads were obtained, of which 74,782 reads passed Albacore QA/QC for 2D sequencing via hairpin and barcode detection. Another 128,460 reads passed Albacore QA/QC but remained unclassified by barcode. Thus, after size selection (3.7–5.7 kb) a total of 65,706 barcoded 2D sequences were screened against a NCBI 16S rRNA gene database by Discontiguous MegaBLAST (see Supplemental Tables S1 and S2). A total of 63,413 sequence yielded a best BLAST hit (BBH) and were rarefied to those BBHs with alignments >1000 bp (54,812 sequences). These sequences (1,566 different BBHs) were parsed and counted for each mouse sample. To determine if the overall gut microbiome differed between the mouse genotypes or activity, a Bray-Curtis dissimilarity matrix was constructed using a fourth root transformation on the BBH sequences^34^ and visualized via non-metric multidimensional scaling (NMDS; Fig. 1). Following a permutational multivariate analysis of variance (PERMANOVA) of the gut microbial community, we found significant differences between physical activity (p = 0.001, R^2^ = 16.6%) among the mice. Host genotype was not significantly different at the community level (p = 0.392, R^2^ = 5.4%). Likewise, the interaction between activity and genotype in mouse fecal samples was not significantly different (p = 0.089, R^2^ = 8.0%).

The dominant bacteria within the fecal samples were determined by averaging across genotype and activity level for the mice and summing the counts for each individual BBHs. Of the top 25 bacterial BBHs within the samples, *Muribaculum intestinale*, *Parasutterella excrementihominis*, *Turicibacter sanguinis*, *Robinsoniella peoriensis*, or *Turicimonas muris* were the most abundant BBHs depending on the particular sample (Supp. Fig. S1). Of note, these preliminary BBH designations represent the best matches of the raw reads to the NCBI 16S rRNA gene database and are not considered definitive identification of any particular microorganism without further phylogenetic testing.

### Enrichment of bacteria by genotype or activity

**Figure 2:**
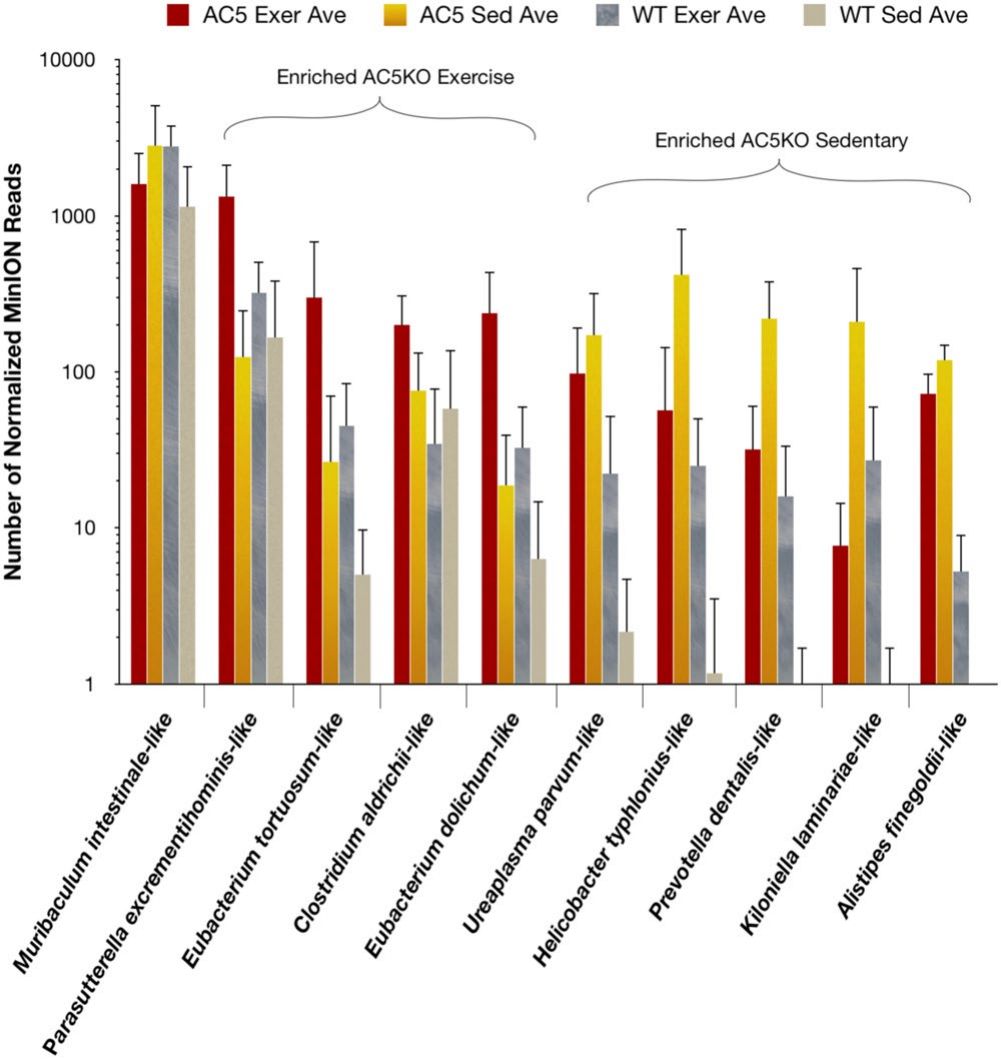
Histogram of best BLAST hits (BBHs) enriched in the AC5KO genotype. Data is presented as Mean + SD. Those particular BBHs more abundant in AC5KO exercise or sedentary mice are indicated.

**Figure 3:**
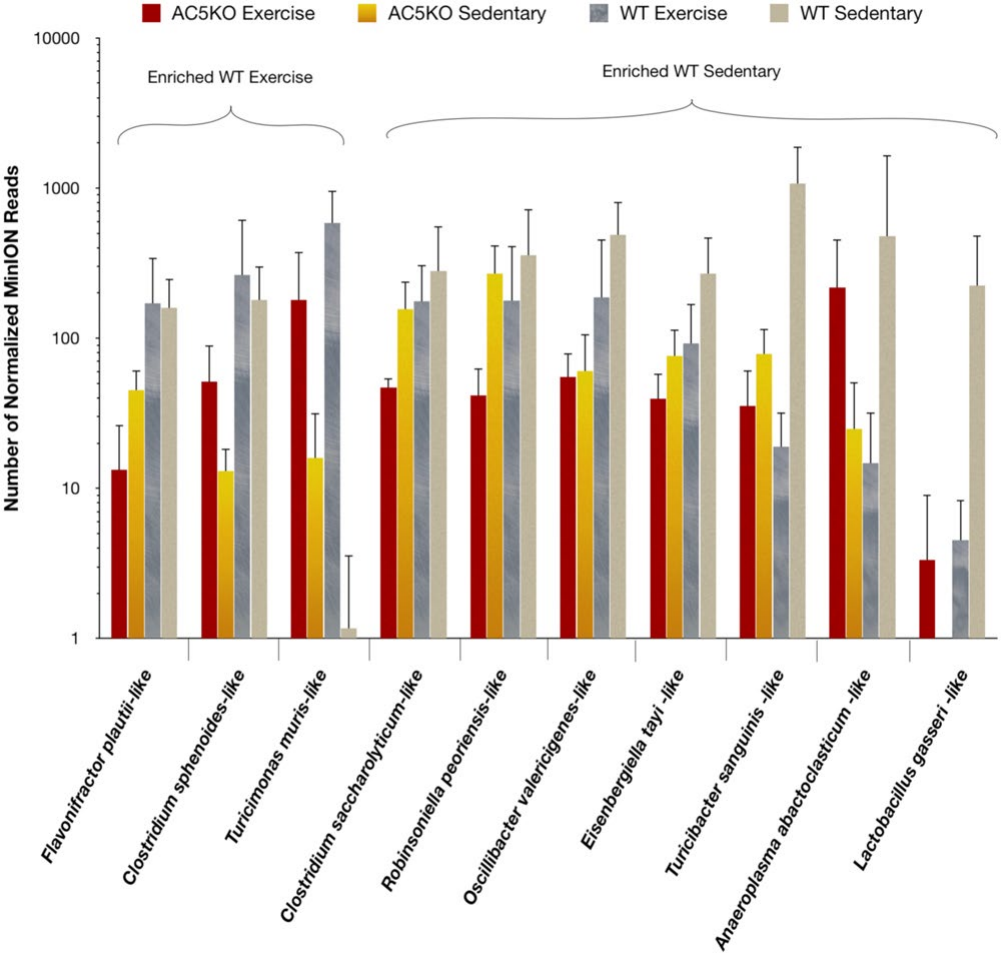
Histogram of best BLAST hits (BBHs) enriched in the Wild Type (WT) genotype. Data is presented as Mean + SD. Those particular BBHs more abundant in WT exercise or sedentary mice are indicated.

To assess those members of the bacterial community that were more prevalent in either the AC5KO or WT mice, the average counts were subtracted from each other to discern the top 10 BBHs enriched within each treatment group. The more abundant BBHs in the AC5KO mice are presented in Fig. 2. BBHs from *Muribaculum intestinale*, *Parasutterella excrementihominis*, two different *Eubacterium* sp., and *Clostridium aldrichii* were enriched in the AC5KO exercise mice. In contrast, BBHs from *Ureaplasma parvum*, *Helicobacter typhlonius, Prevotella dentalis*, *Kiloniella laminariae*, and *Alistipes finegoldii* were more prevalent in the AC5KO sedentary mice. The gut bacteria dominating in WT mice are presented in Fig. 3. BBHs identified *Turicimonas muris, Clostridium sphenoides* and *Flavonifractor plautii* were more abundant in the WT exercise mice, while BBHs corresponding to *Clostridium saccharolyticum*, *Robinsoniella peoriensis*, *Oscillibacter valericigenes, Eisenbergiella tayi, Anaeroplasma abactoclasticum*, *Turicibacter sanguinis*, and *Lactobacillus gasseri* were most abundant in the WT sedentary mice.

### Assessing if AC5KO or WT mice harbor unique bacterial BBHs depending on mouse activity levels

**Figure 4:**
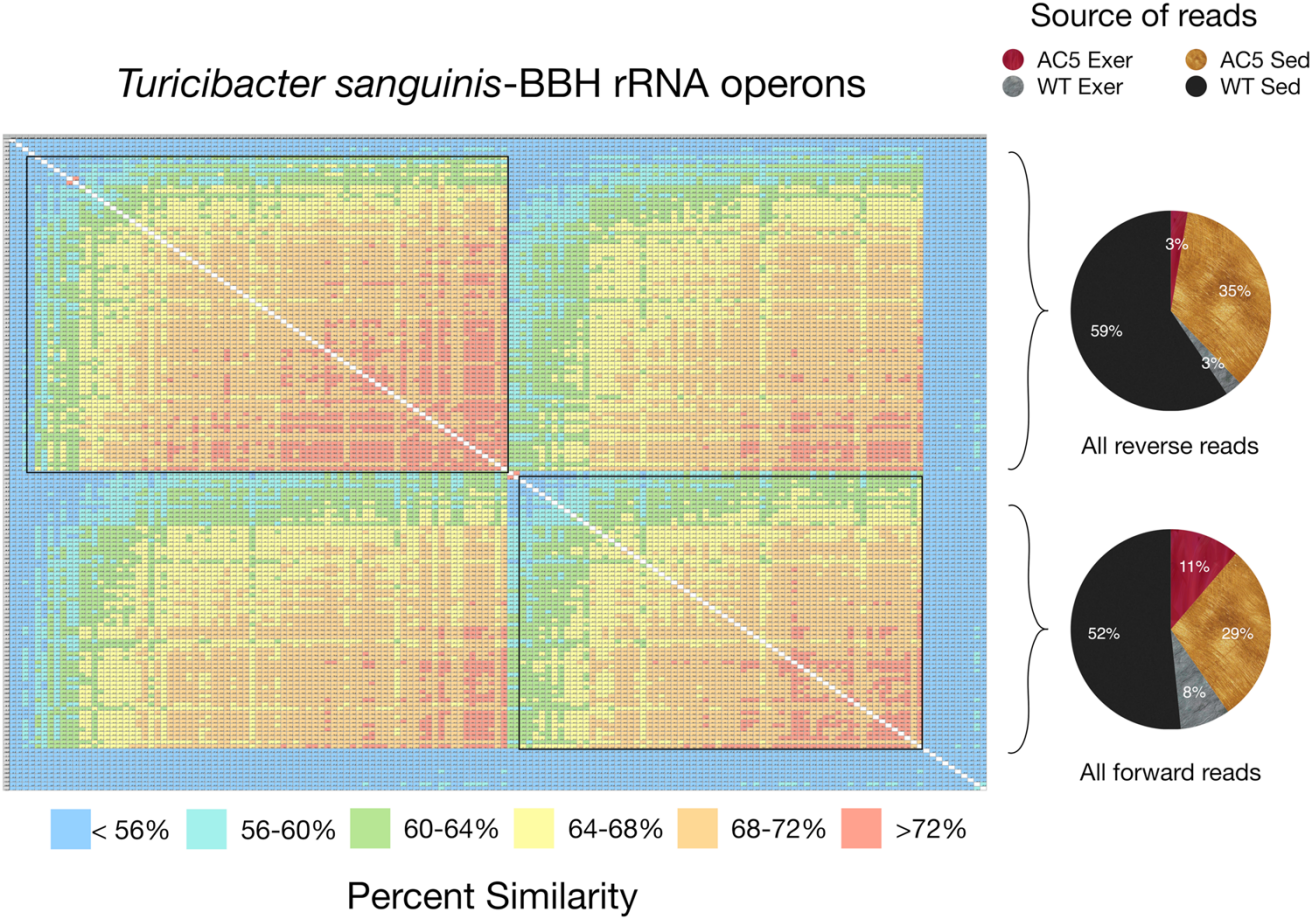
Heatmap of aligned *Turicibacter sanguinis* sequence reads from the top 10–20 matches from the various biological treatments (i.e. AC5KO vs. WT vs. exercise vs. sedentary). The alignment is grouped by similarity and the color code displaying percent similarity is detailed in the legend below. The pie charts on the right indicate the source of the reads corresponding to the brackets in the alignment.

**Figure 5:**
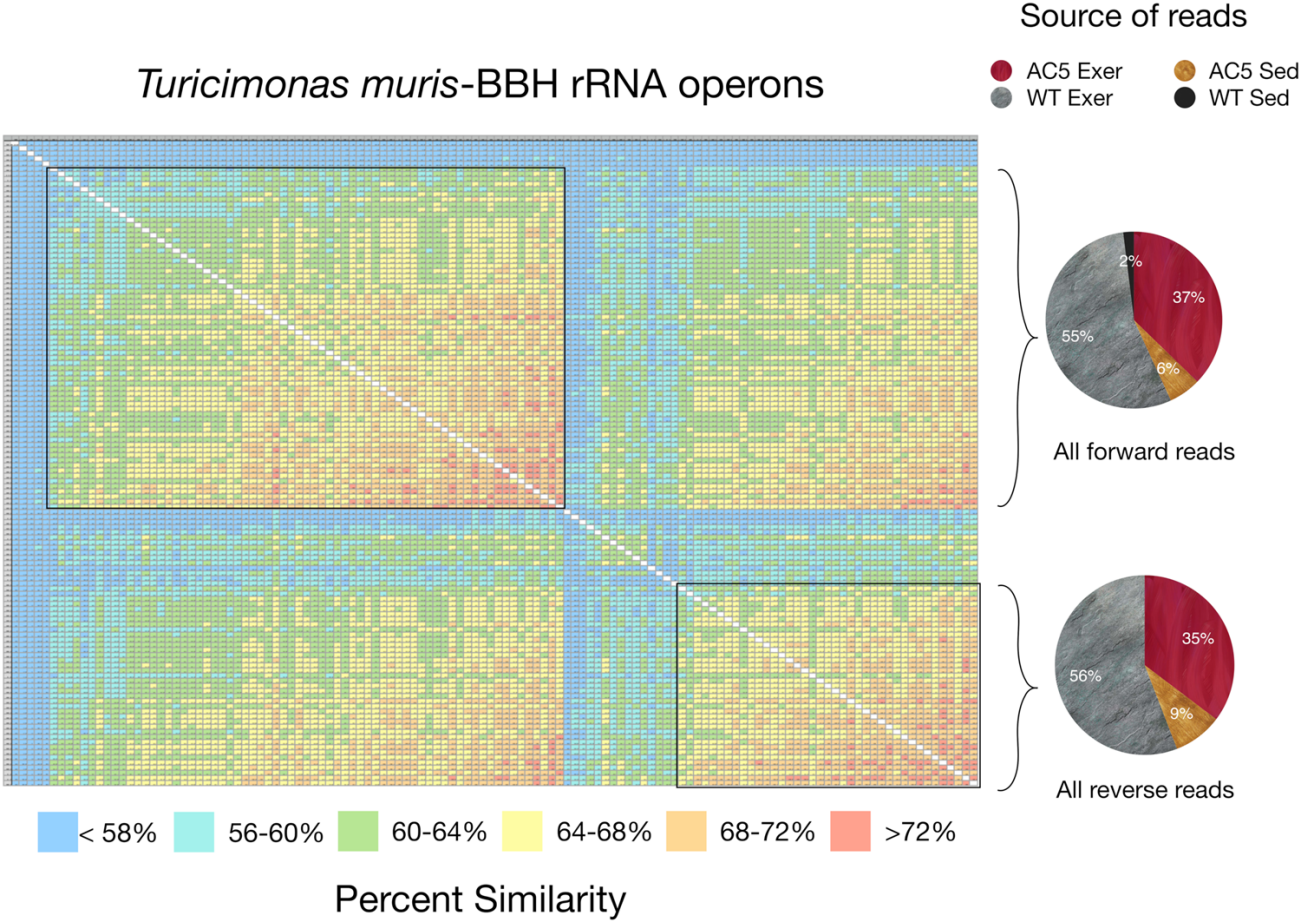
Heatmap of aligned *Turicimonas muris* sequence reads from the top 10–20 matches from the various biological treatments (i.e. AC5KO vs. WT vs. exercise vs. sedentary). The alignment is grouped by similarity and the color code displaying percent similarity is detailed in the legend below. The pie charts on the right indicate the source of the reads corresponding to the brackets in the alignment.

In order to discern if AC5KO or WT mice harbored unique microbiota, the top 10–25 BBH reads (highest alignment length/similarity score) from the mouse samples were aligned; ordered by similarity; a distance matrix created; and the values presented as a heatmap. If all genotypes and activity levels harbored the same bacterial species/strain, the heatmap would demonstrate a uniform shift from high similarity to lower similarity (reflecting MinION sequencing error) with source reads for the alignment equally distributed among the various mice genotypes/activity levels. However, if the various mouse genotypes/activity levels harbored unique bacterial species, then the heatmap would display distinct clusters of sequences that were unevenly distributed from the different groups of mice. Interestingly, a slight variation of this expected pattern was observed for the BBHs corresponding to *Turicibacter sanguinis* (Fig. 4) and *Turicimonas muris* (Fig. 5). Two major clusters can be seen along the diagonal with strong similarity between the clusters. When analyzing for the source of the reads generating these clusters, all mouse treatments are represented, suggesting highly similar raw sequence reads for this particular BBH from all mice. Interestingly, the upper cluster for *Turicibacter sanguinis* represents 2D reads all generated in the reverse direction while the lower cluster are reads all generated in the forward direction (Fig. 4). This patterning suggests that the MinION does not display a uniform error rate along the sequence read for the particular flow cell and sequencing kit used in this study. That is, the reverse and forward reads can be resolved based on similarity alone. A comparable pattern is observed with the *Turicimonas muris* BBH with 2 main clusters distributed among the various mouse samples but grouped by sequence direction with respect to the rRNA operon (Fig. 5).

**Figure 6:**
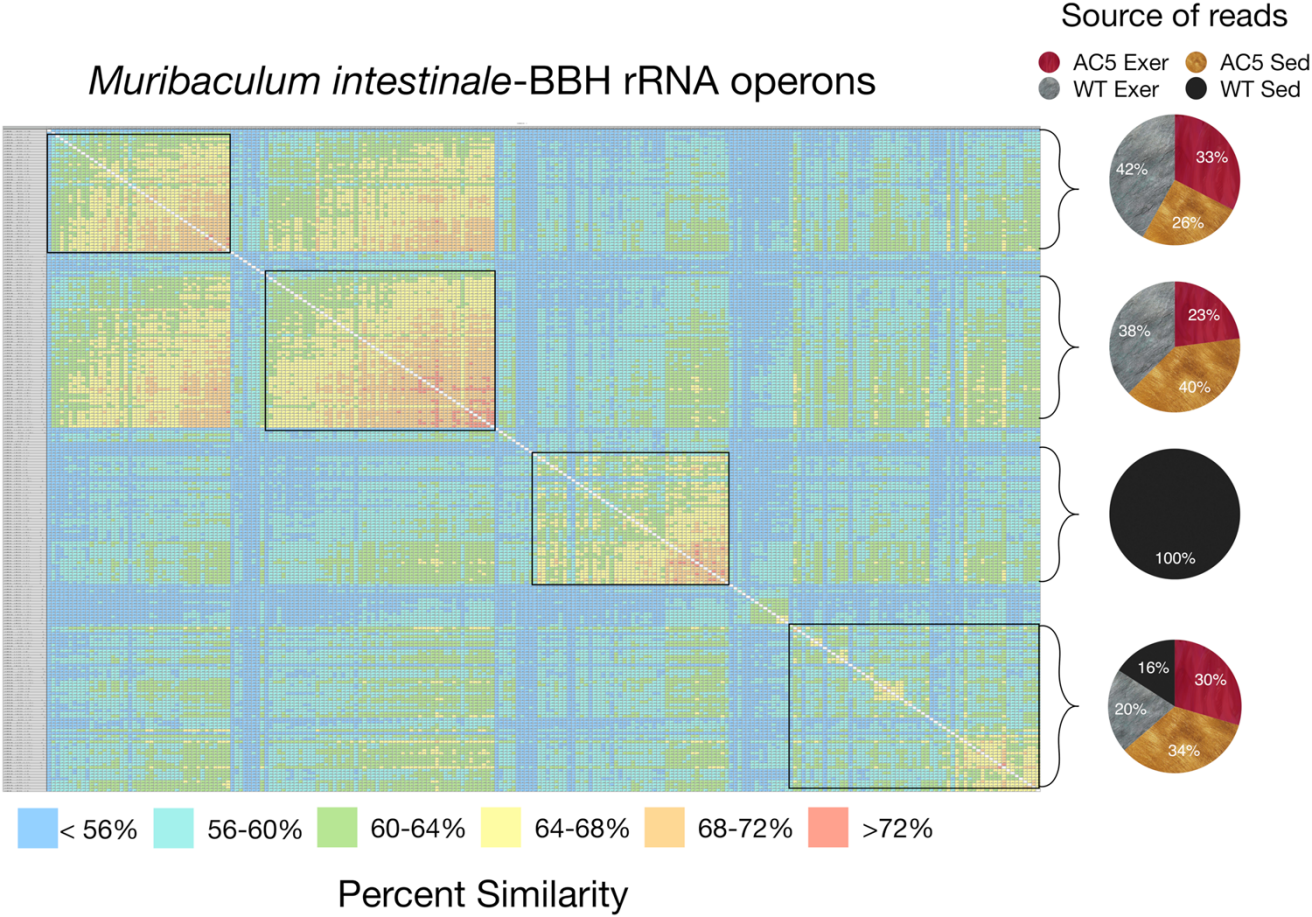
Heatmap of aligned *Muribaculum intestinale* sequence reads from the top 15 matches from the various biological treatments (i.e. AC5KO vs. WT vs. exercise vs. sedentary). The alignment is grouped by similarity and the color code displaying percent similarity is detailed in the legend below. The pie charts on the right indicate the source of the reads corresponding to the brackets as indicated.

**Figure 7:**
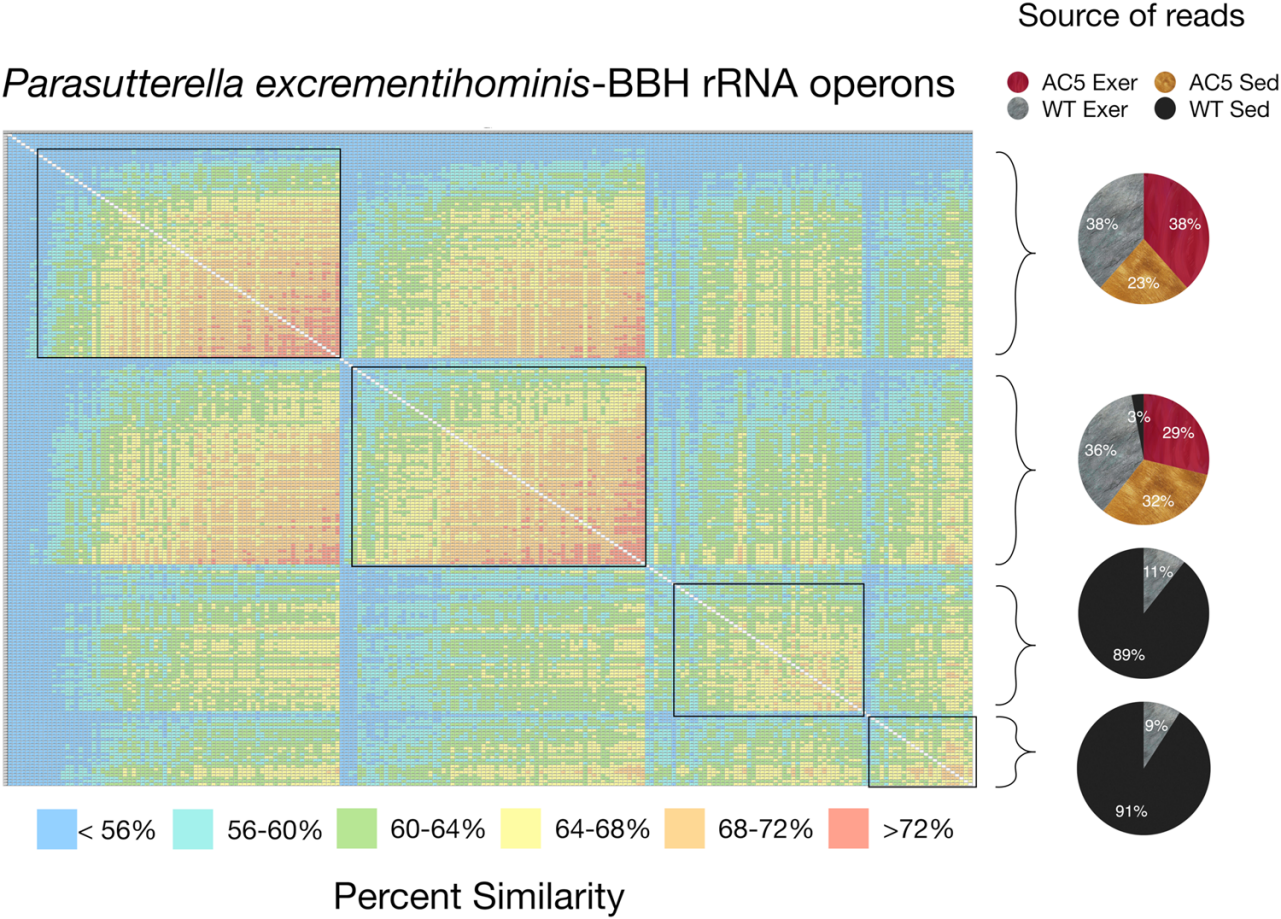
Heatmap of aligned *Parasutterella excrementihominis* sequence reads from the top 15–20 matches from the various biological treatments (i.e. AC5KO vs. WT vs. exercise vs. sedentary). The alignment is grouped by similarity and the color code displaying percent similarity is detailed in the legend below. The pie charts on the right indicate the source of the reads corresponding to the brackets in the alignment.

In contrast, the heatmaps for the BBHs corresponding to *Muribaculum intestinale* or *Parasutterella excrementihominis* are much more structured (Figs. 6 and 7). For the *M. intestinale*-like reads, the heatmap contains 4 distinct clusters along the diagonal and these clusters are grouped by the source of the raw reads (Fig. 6). The top two clusters are nearly equally represented by reads from the AC5KO and WT exercise mice in the forward and the reverse read direction, while the third cluster is exclusive to WT sedentary mice in both read directions. The bottom cluster is also represented by sequences from all mice in both directions. These clusters contain sequences that are more similar to each other than the associated sequences from mice with different genotypes/activity levels. A similar clustering based upon read direction or source of reads was also demonstrated for other bacterial species (Supplemental Figs. S2 and S3).

### Species selection by AC5KO or WT mice with activity level

**Figure 8:**
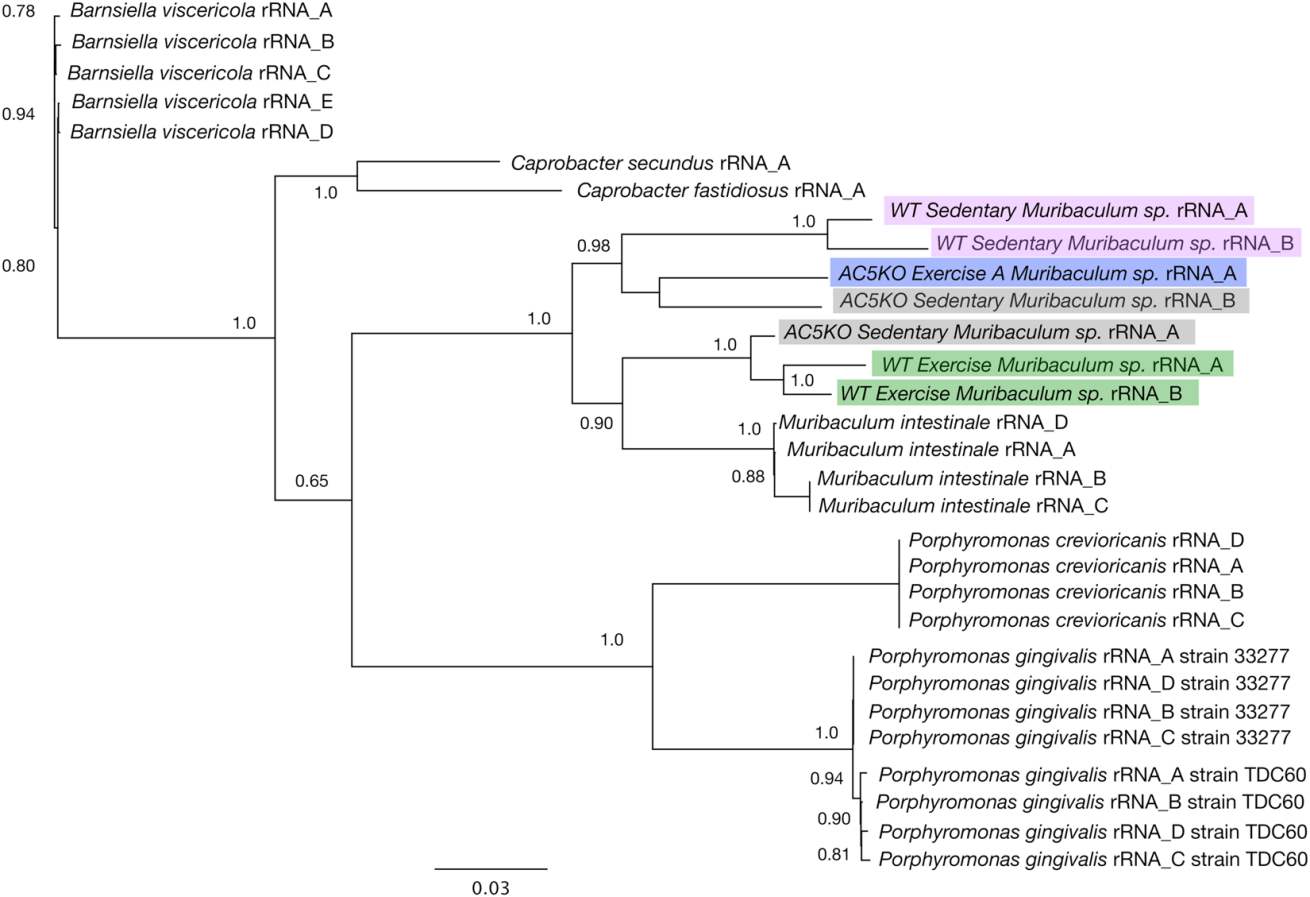
Phylogenetic tree reconstruction of various *M. intestinale* consensus rRNA operons using FastDNAML from the AC5KO and WT mice compared with rRNA operons from other Bacteroidales genomes. Note that each sequenced genome contains 4–5 rRNA operons. The bootstrap support for the tree topology is indicated. The tree is based on 2952 unambiguously aligned base pairs from the 16S/23S rRNA genes.

**Figure 9:**
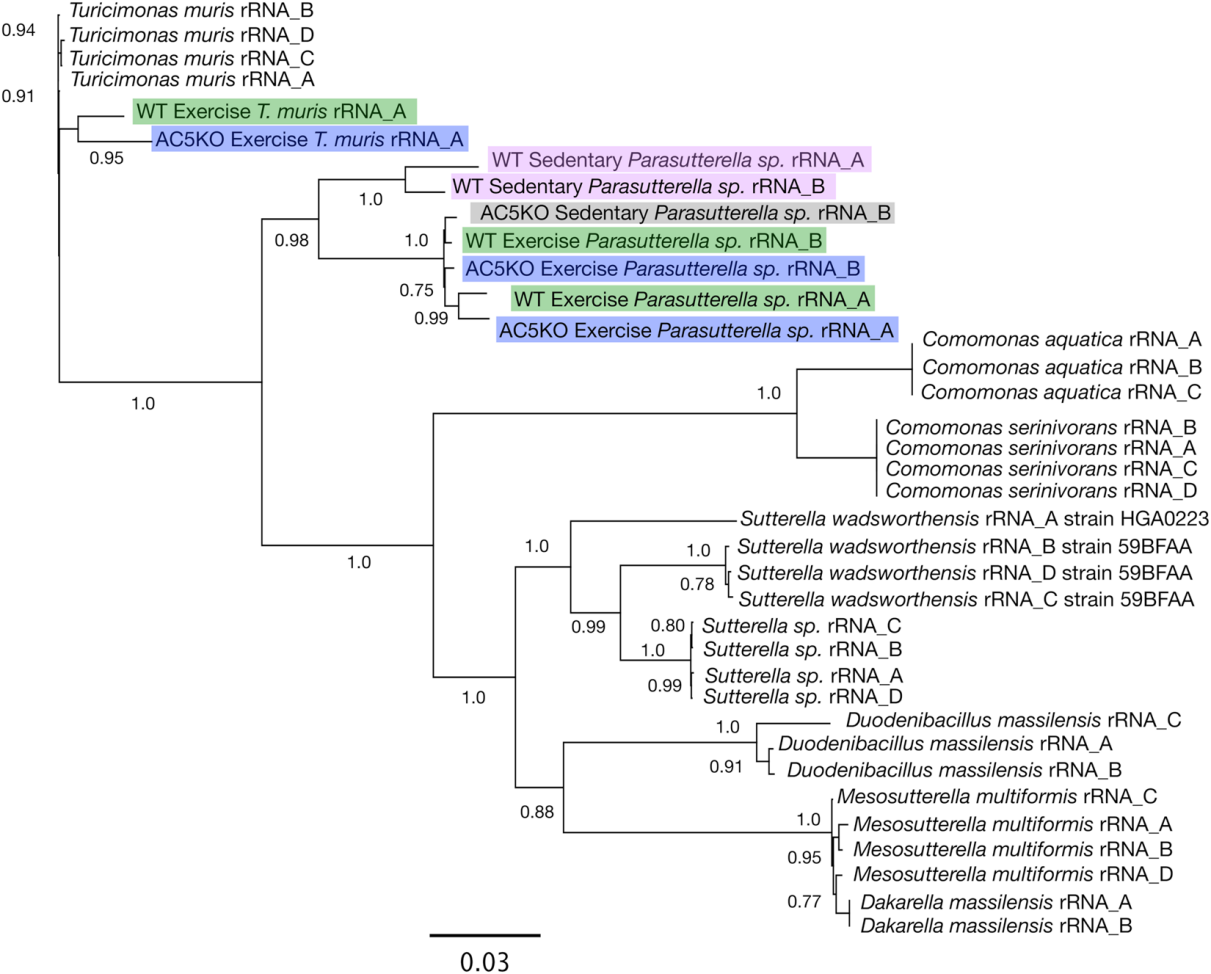
Phylogenetic tree reconstruction of various *P. excrementihominis* and *T. muris* consensus rRNA operons using FastDNAML from the AC5KO and WT mice compared with rRNA operons from other Burkholderiales genomes. Note that each sequenced genome contains 4–5 rRNA operons. The bootstrap support for the tree topology is indicated. The tree is based on 3760 unambiguously aligned base pairs from the 16S/23S rRNA genes.

In order to determine if the clustering within the heatmaps represented different species of the *M. intestinale*-like or the *P. excrementihominis*-like BBHs in the various fecal samples, it was necessary to reconstruct rRNA operons from the raw reads from the different sample sets; align to complete rRNA operons from different species within the genus (or closely related taxa); build phylogenetic trees that resolve the strains of these known taxa; and assess if these trees also resolve the rRNA operons from the AC5KO or WT strains obtained in this study. Tree reconstruction of the *M. intestinale*-like consensus rRNA operons and associated taxa is shown in Fig. 8. The tree is based on 2,952 unambiguously aligned base pairs from the 16S and 23S rRNA genes. From the tree, it is clear that all 4 copies of the rRNA operons of the two reference *Porphyromonas* species and strains are resolved within this data set. Likewise, all operons from any particular taxon are closely associated with all other rRNA operons from within that same taxon. In addition, all consensus rRNA operons from the AC5KO and WT type mice that are identified as *M. intestinale* in our data analysis pipeline cluster with *Muribaculum intestinale* strain YL27 from a C57BL/6J wildtype mouse^35^. However, the *M. intestinale* rRNA operons from WT sedentary mice are distinct from all others in this study and confirm the findings shown in the NMDS plot (Fig. 1) and the heatmap (Fig. 6). Likewise, the *M. intestinale* consensus operons from WT exercise mice and the AC5KO mice are distinct. In addition, the phylogenetic tree of *P. excrementihominis* and associated taxa resolve strains of *Sutterella wadsworthensis* and other species of related Betaproteobacteria (Fig. 9) where again, the rRNA operons from WT sedentary mice form a distinct cluster confirming the heatmap clustering of these raw reads.

To determine if AC5KO and WT mice might harbor new bacterial species related to *M. intestinale*, *P. excrementihominis* and *Turicimonas* sp., the 16S rRNA genes were aligned and compared to near-full length and shorter, variable regions of related taxa (available in Genbank) as shown in Supplemental Fig. S6. For the *Muribaculum* associated operons, the similarity matrix for the near-full length 16S rRNA gene suggests that all mice contain a different *Muribaculum* species in the gut since none of the 16S rRNA genes has above 97% similarity to *M. intestinale*. Interestingly, the *Muribaculum sp*. in AC5KO sedentary and WT exercise mice are 98% similar, consistent with the nMDS analysis (Fig. 1). For *Parasutterella* spp., the WT sedentary mice harbor *P. excrementihominis* while the other mouse groups clearly contain a different *Parasutterella* species. Finally, the WT and AC5KO exercise mice harbor a *Turicimonas* sp., yet their 16S rRNA genes differ between each other at 96% similarity, suggesting two distinct species. Phylogenetic analysis of the shorter reads from *Muribaculum*, *Parasutterella*, and *Turicimonas* generate many of the same patterns observed with the near full-length 16S rRNA gene but demonstrate a higher overall similarity, which may lead to incorrect identification and the inability to differentiate between some closely related species. For example, the 16S rRNA genes from WT exercise mice are 97% similar to *M. intestinale* when the analysis is made with a 275 bp aligned region, while the near full length 16S rRNA gene (1330 bp) was 94% similar.

## Discussion

The advent of high-throughput sequencing has allowed for rapid investigations into diverse host-associated and environmental microbiomes^36,37^. For example, several studies have shown that factors such as diet/exercise/genotype or toxin exposure differentially alter the mammalian gut microbiome^10,13,14,16,18,38–40^. Nearly all of these studies of gut bacterial microbiota, have reported at the genus through phylum level using the V1–V6 variable regions of the 16S rRNA gene^1,3,38,41,42^. For instance, many groups have demonstrated that *Muribaculum intestinale* (formerly designated as family 24–7) is a dominant member of the mouse gut bacterial community^10,21,35,43^. Likewise, *Alcaligenes* spp. or *Clostridium* spp. are detected as dominant members of the mouse gut microbiome but are often not resolved at the species level. In contrast to the V1–V6 methods cited above, MinION profiling of rRNA operons provides 10–100× longer reads, albeit with an increased error rate. However, accurate OTU calling at ~80% identity for long 16S rRNA gene reads, no detectable chimeras, species-level resolution, and a quantitative capability has been previously demonstrated for the MinION for over 100 of the numerically-abundant rRNA operons of complex communities^24^. Furthermore, these longer MinION raw reads can be used to generate a robust consensus sequence for the rRNA operons which provide far greater taxonomic resolution than shorter fragments of the 16S rRNA gene alone. This long read capability of the MinION has been employed in a wide variety of studies from the human genome^19,44,45^, human pathogens^46,47^, antibiotic resistance^48,49^ and for metagenomics in various environments^24,50,51^.

In this report, we used near full-length rRNA operon reads and consensus building to characterize the gut microbiome from mice with different genotypes and physical activity levels. Our data demonstrated resolution of different *Muribaculum* and *Parasutterella* spp. within the AC5KO/WT exercise mice and WT sedentary mice, while *Turicibacter* and *Turicimonas* species were the same between genotype and activity level. Our findings are consistent with a previous study that compared Illumina short reads with MinION long reads for characterization of the mouse gut community and reported no significant differences in major taxonomic units (89%) between the platforms, with the MinION demonstrating species-level resolution of the microbiome^21^.

The role that specific dominant bacterial species may play in mouse gut physiology has yet to be determined. *M. intestinale* is reported to be strictly anaerobic and capable of galactose degradation^35^ or associated with homoserine or serine metabolism^43^. Additionally, *M. intestinale* inhibited colonization by a known human pathogen, *Salmonella enterica* serovar Typhimurium in a mouse model^52^. *Parasutterella excrementhominis* is also a strict anaerobe and may be a short chain fatty acid producer in the gut^53^. Interestingly, other bacteria detected in our mouse models are considered pathogens. For example, a dominant bacterium within AC5KO sedentary mice is *Helicobacter typhlonius*, thought to be involved with inflammatory bowel disease^54^. Another abundant bacterium in WT sedentary mice (*Turicibacter sanguinis* MOL361) was isolated from a febrile patient with acute appendicitis^55^. Likewise, *Alistipes finegoldii* was isolated from a patient with colon cancer^56^ and was enriched in the most frail, middle-aged or old mice in a study on aging in the gut^9^. Other potential pathogens in the AC5KO mice include *Ureaplasma parvum* which is correlated with infertility or *Prevotella dentalis* associated with gum disease. Yet, AC5KO mice are completely asymptomatic for any of these dysbiotic conditions.

Paradoxically, several taxa thought to improve host health were only present in very low abundance in AC5KO or were undetectable in WT mice. One of the most abundant anaerobes in the human gut, *Faecalibacterium prausnitzii*^57^, is believed to play a pivotal role in a healthy gut^58^. We could detect only 2 reads corresponding to *F. prausnitzii* out of over >4500 total reads in the AC5KO exercise mice. Another beneficial gut microbe suspected to improve host health is *Akkermansia muciniphila*^17,59,60^ which was also only observed in 2 reads for the AC5KO exercise mice and not detected in any WT mice or AC5KO sedentary mice. Additionally, AC5KO exercise mice demonstrated reduced rarefaction and the lowest Chao1 diversity (Supp. Figs. S4 and S5B). These findings of suspected pathogens in high abundance and the lack of known beneficial microorganisms in AC5KO and WT exercise mice highlights the need to consider gut microbes as more than just a single physiological or clinical type. Bacteria typically contain 2500–5000 different genes (including pathogenicity islands) which are not all expressed simultaneously. The thought that “pathogens” may also be providing a beneficial service to hosts under the right conditions, when different genes are being expressed, is consistent with our findings of *Helicobacter typhlonius* and *Alistipes finegoldii* in asymptomatic AC5KO sedentary mice. Furthermore, Zhao et al. has discussed the importance of considering guild-based analysis—as opposed to taxon-based investigations—as a more ecologically relevant means to improve the identification of functionally distinct members of the gut microbiota in the context of both human health and disease^61,62^. It is also important to note that our findings are in line with reports of stochastic effects associated with rearing conditions^63^. Since the AC5KO and WT mice have been reared at the Rutgers New Jersey Medical School Animal Facility for over 15 years, it is not surprising that the gut microbiome differs from earlier reports of WT animals obtained from commercial vendors (e.g., Taconic or Jackson Labs). Finally, given the small number of AC5KO and WT mice in this report, the characterization of the gut microbiome should be validated with a larger cohort of mice.

## Conclusions

As life expectancy has increased, the need to understand all aspects of healthful aging to promote longevity is paramount. The AC5KO mouse model and the increased resolution of rRNA operon profiling by MinION sequencing uniquely allows for exploration of multiple aspects of healthful aging, with a central focus on the contribution of individual microbes on the phenotype of the host. Once specific strains have been obtained by culturing efforts and further physiological analyses completed, it may be possible to design new diagnostic tools and improved therapeutic agents with the potential to deliver particular compounds (i.e. short chain fatty acids, proteins), drugs or gene therapy vectors based on knowledge of bacterial species/strains within the gut to treat the global rise of chronic disease.

## Methods

### Animal experimental procedures

All experiments were performed on 3-month old AC5KO mice and their matched WT littermates (by body weight) which have been housed/bred at Rutgers New Jersey Medical School for over 15 years. All AC5KO and WT (originally C57BL/6J from Jackson Labs; Farmington, CT) mice were males, with the exception of a single AC5KO female in the exercise cohort. As littermates, AC5KO and WT mice have the same dam, the same environmental conditions during rearing, and the same maternal microbial exposure during weaning. Furthermore, all animals were provided the same standard chow and had *ad libitum* access to food and water for the length of the study in accordance with the *Guide for the Care and Use of Laboratory Animals* (National Research Council, Eighth Edition 2011). After weaning (21 days), the mice were placed in separate cages (4–5 per cage) based on genotype in the same animal facility. The mice were then acclimatized for 9 weeks prior to baseline exercise determinations. Mice were exercised using a treadmill (Exer-6M, Columbus Instruments). After exercise capacity was assessed, the AC5KO and WT mice were then randomly assigned to either an exercise or sedentary treatment. For the first 2 weeks of exercise training, the running speed was gradually increased until the mice could attain the desired workload (70% of maximal speed, 1 hour/day, 5 days/week for an additional 3 weeks). After a total of 5 weeks of exercise training, mice were sacrificed using previously described protocols^30^. Sample sizes for this study were: AC5KO exercise, n = 3; AC5KO sedentary, n = 4; WT exercise, n = 4; WT sedentary, n = 6.

### Bacterial DNA extractions and purification

Animal fecal samples were collected from the distal colon, snap frozen in liquid nitrogen, and stored at −80 °C until extraction. DNA was extracted using a modified CTAB phenol/chloroform extraction method. Briefly, 50 μl of a buffer solution (50 mM Tris, 10 mM EDTA, 0.7 M NaCl) was added to the fecal material and the sample was subjected to 5 rapid freeze/thaws. The samples were then mixed with 450 µl of the buffer containing 0.1 g of cetyltrimethyl ammonium bromide (CTAB) and 10 µl β-mercaptoethanol. The mixture was extracted twice with 800 μl phenol-chloroform and ethanol precipitated. The DNA extracts were further purified with an Agencourt AMPure beads (Beckman Coulter, Brea, CA, USA) cleanup using 15 µl DNA extract and combined with 15 μl of sterile DI water, 15 µl of AMPure beads, 15 µl 5 M NaCl, and 15 µl 30% PEG/1.5 M NaCl. The DNA was bound to beads for 15 min, washed 2× with 70% ethanol, resuspended in 15 µl of 10 mM Tris buffer, and stored at −80 °C until used for PCR.

### rRNA operon amplifications

Near full-length bacterial ribosomal operons were amplified with the 16S rRNA-27Forward primer and the 23S rRNA-2241Reverse primer, <10 ng template DNA, and a Hi-Fidelity Taq polymerase (Bimake LLC, Houston, TX, USA) as described in^24^. Ribosomal operons were amplified via touchdown PCR: Initial denaturation 5 min at 95 °C; 2 cycles of 95 °C/20 sec for denaturation, 68°/15 sec for primer annealing, 72°/ 75 sec for extension; then 2 cycles of 66 °C for primer annealing; 2 cycles of 64 °C for primer annealing; 2 cycles of 62 °C for primer annealing-all with denaturation/extension; followed by 22 cycles of denaturation, 60 °C/15 sec for primer annealing, extension; and a final extension at 72 °C for 5 min. At the end of the 16^th^ cycle (8 touchdown + 8 standard cycles), 12 μl of amplification mixture was removed and stored at −80 °C. The amplification was allowed to proceed until 30 cycles was completed and the PCR product was visualized by agarose gel electrophoresis.

Following verification of successful amplification by agarose gel electrophoresis, the 16 cycle PCR products were purified by AMPure bead clean-up as described above and a second round of amplification was initiated using modified overhang primers for barcoding (i.e. 16S rRNA-27Forward OH primer: 5′-TTT CTG TTG GTG CTG ATA TTG C-[ONT barcode overhang for PCR labeling]-AGA GTT TGA TCC TGG CTC AG-3′) and (23S rRNA-2241Reverse OH primer: 5′-ACT TGC CTG TCG CTC TAT CTT C–[ONT barcode overhang for PCR labeling]-ACC GCC CCA GTH AAA CT-3′). The 16 cycle OH product was captured, as above, and bead purified before final barcode amplification using the ONT barcoding kit. Barcode amplification conditions were 5 min at 95 °C, followed by 30 cycles of 95 °C for 20 sec, 60 °C for 15 sec and 72 °C for 1:15 sec, followed by extension cycle at 72 °C for 5 min. Barcoded rRNA amplicons were visualized and quantified by agarose gel electrophoresis.

### Library preparation and sequencing by MinION

MinION library construction employed the 2D sequencing kit. Twelve barcoded amplicons (1200 ng total) were combined, end-repaired, dA-tailed as per ONT instructions using NEB kits (New England Biolabs, Ipswich, MA, USA) and the modified AMPure bead purification described above. Ligation of the ONT adaptor and hairpin employed the Blunt/TA ligase master mix (NEB) with an addition of 1 µl of freshly-prepared ATP solution (~4 mg/ml) to facilitate ligation. All libraries were analyzed on R9 flow cells.

### Data analysis

MinION fast5 files were basecalled, separated by barcode, and converted to fastq files using Albacore (2.1). Raw reads between 3700–5700 bp in length from each sample were screened against an NCBI rRNA database (Bioproject 33175) to determine best BLAST hits (BBH) using Discontiguous MegaBLAST^24^. Settings included a word size of 11, gap cost of 5/2, scoring of 2/−3, and a seed length of 18. The top hit output was exported as .csv files, counted, and normalized across the different samples by multiplying with a conversion factor to generate identical total reads for all samples. The read numbers were then averaged across genotype and activity status to determine dominant members of the gut bacterial community. Enrichment histograms were constructed by subtracting total average raw read numbers from either genotype or physical activity to determine the top BBHs within each cohort. Averages/standard deviations were calculated on those enriched BBHs based on genotype or exercise level.

### NMDS and diversity analysis

Raw reads were copied into Excel and individual species counts were calculated from each sample (n = 17) creating a community data matrix. The vegan package^64^ was used in R (3.6.1) to compute a fourth root transformation of the raw data and create a dissimilarity matrix using a pairwise Bray-Curtis dissimilarity measure^34^. NMDS plots were created from the Bray-Curtis dissimilarity matrixes^65^. PERMANOVA using the vegan ‘adonis’ code was performed to test for significance. PAST3^66^ was used to determine individual rarefaction curves and measures of alpha diversity (Chao1 & Shannon diversity indexes) using normalized read counts.

### Distance matrices and heatmaps

To assess if any particular BBH displayed heterogeneity across genotype or activity distance matrices were created by pair-wise comparison of OTU reads from each treatment. To ascertain if genotype or activity level exhibited clustering for the enriched OTUs, the top 15–25 raw reads from each mouse sample (based on percent similarity/alignment length) were extracted, annotated with 16S rRNA universal primers (e.g. 27F, 343F, 519F, 907F, 1392F, and 1492F- at 85% identity), oriented in the same direction, and aligned using MUSCLE across all treatments. This alignment was then grouped based on similarity rather than file input order. Any reads missing primer annotation were discarded from further analysis. The distance matrix was generated from these similarity-ordered alignments, exported, and colorized for ease of visualization in Excel or Numbers. Clusters (if they existed) were identified visually.

### Operon reconstruction

Consensus rRNA operons were reconstructed using an iterative approach and the LastZ alignment tool^24^. Because many of the bacteria identified in this study contained multiple rRNA operons, it was necessary to first align all raw reads of a particular BBH, annotate the tRNAs (alanine or isoleucine) or assess the length polymorphisms within the ITS region, and then group reads by these additional sequence features prior to reconstructing the consensus rRNA operons. Since, there were large differences in the number of raw reads for a particular BBH from a particular mouse, it was not always possible to reconstruct rRNA operons with adequate coverage. Additionally, the sequence variability in the raw reads from BBH’s with some mice prevented the reconstruction of a robust rRNA consensus.

### Phylogenetic trees and Species-level verification

Once the rRNA operons were re-constructed (termed rRNA_A or rRNA_B), the consensus AC5KO and WT rRNA operons were aligned with rRNA operons from closely associated taxa for which complete or near complete genomes are available in the NCBI database. Phylogenetic tree reconstruction was based on unambiguously aligned 16S and 23S rRNA gene sequences as indicated in the figure legends using FastDNAML with 100 bootstrap replications in Geneious 11.1.3.

### Ethics approval

All animals were maintained in accordance with the *Guide for the Care and Use of Laboratory Animals* and protocols approved by the IACUC of Rutgers University and the New Jersey Medical School.

## Supplementary information

Supplemental information.

Dataset 1.

## Data Availability

All data is currently being made available at NCBI SRA (BioProject # PRJNA529518).
