# Supplementary material for: Host genotype and exercise exhibit species-level selection for members of the gut bacterial communities in the mouse digestive system
Source: Sci Rep. 2020 Jun 2;10:8984. doi: 10.1038/s41598-020-65740-4 (PMC7265280; doi:10.1038/s41598-020-65740-4)
Supplement: Supplementary file 1 — Supplemental information. [file 41598_2020_65740_MOESM1_ESM.pdf]

Host genotype and exercise exhibit species-level selection for members of the gut bacterial communities in the mouse digestive system

Supplemental Materials:

RA Dowden [1], LR McGuinness [2], PJ Wisniewski [1], SC Campbell [1], JJ Guers [3], M Oydanich [4], SF Vatner [4], MM Häggblom [5], LJ Kerkhof [2*]

[1] Department of Kinesiology and Health, Rutgers, the State University of New Jersey, New Brunswick, NJ 08901

[2] Department of Marine and Coastal Sciences, Rutgers, the State University of New Jersey, New Brunswick, NJ 08901

[3] Department of Biology, Behavioral Neuroscience, and Health Sciences, Rider University, Lawrenceville, NJ 08648

[4] Department of Cell Biology and Molecular Medicine, Rutgers, the State University of New Jersey, Newark, NJ

[5] Department of Biochemistry and Microbiology, Rutgers, the State University of New Jersey, New Brunswick, NJ 08901

*Corresponding Author: LJK

lkerkhof@rutgers.edu

Supplemental Fig. 1. Pie chart of relative abundance for the dominant bacteria observed in this study (n=24 taxa).

Supplemental Fig. 2. Heatmaps of aligned sequence reads from the top 5-20 matches from the various biological treatments (i.e. AC5KO vs WT vs exercise vs sedentary) for the OTUs indicated. The alignment is grouped by similarity and the color code displaying percent similarity is detailed in the legend below. The pie charts on the right indicate the source of the reads corresponding to the brackets in the alignment.

Supplemental Fig. 3. Heatmaps of aligned sequence reads from the top 5-20 matches from the various biological treatments (i.e. AC5KO vs WT vs exercise vs sedentary) for the OTUs indicated. The alignment is grouped by similarity and the color code displaying percent similarity is detailed in the legend below. The pie charts on the right indicate the source of the reads corresponding to the brackets in the alignment.

Supplemental Fig. 4. Rarefaction curve of average reads for the mouse models as indicated.

Supplemental Fig. 5. **A**: Shannon and **B:** Chao1 diversity indices from normalized read averages from AC5-EX, AC5-SED, WT-EX & WT-SED. Values shown with 95% confidence intervals.

Supplemental Fig. 6. Heat maps displaying similarity among short and long reads of the 16S rRNA genes to the taxa identified in the Discontinuous MegaBlast searches of the MinION operon profiling.

Supplemental Table 1. Sequencing statistics using the MinION platform.

Supp. Fig. 1

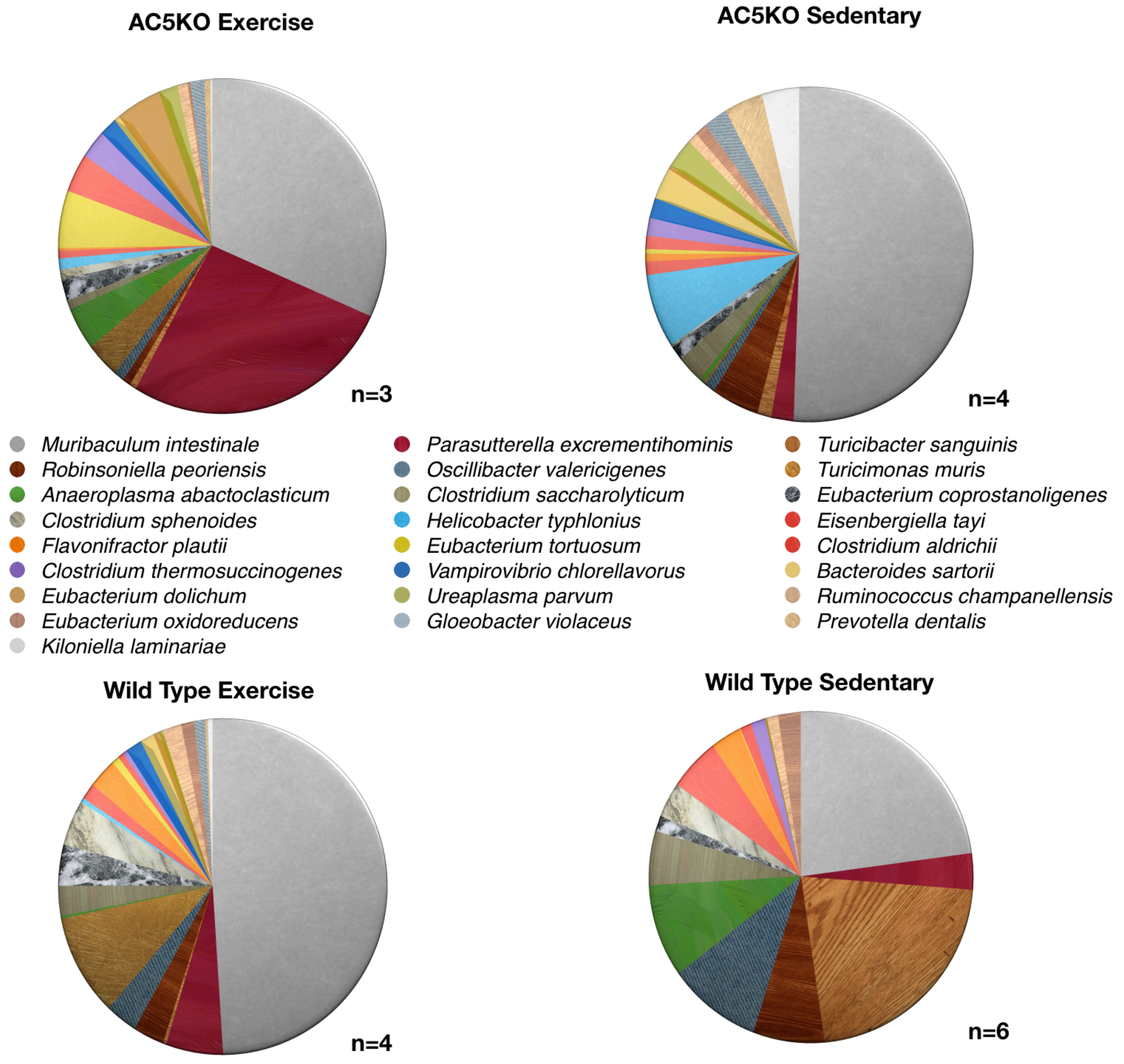
AC5KO Exercise
n=3
AC5KO Sedentary
n=4
Muribaculum intestinale
Robinsoniella peoriensis
Anaeroplasma abactoclasticum
Clostridium sphenoides
Flavonifractor plautii
Clostridium thermosuccinogenes
Eubacterium dolichum
Eubacterium oxidoreducens
Kiloniella laminariae
Parasutterella excrementihominis
Oscillibacter valericigenes
Clostridium saccharolyticum
Helicobacter typhlonius
Eubacterium tortuosum
Vampirovibrio chlorellavorus
Ureaplasma parvum
Gloeobacter violaceus
Turicibacter sanguinis
Turicimonas muris
Eubacterium coprostanoligenes
Eisenbergiella tayi
Clostridium aldrichii
Bacteroides sartorii
Ruminococcus champanellensis
Prevotella dentalis
Wild Type Exercise
n=4
Wild Type Sedentary
n=6

Supp. Fig. 2

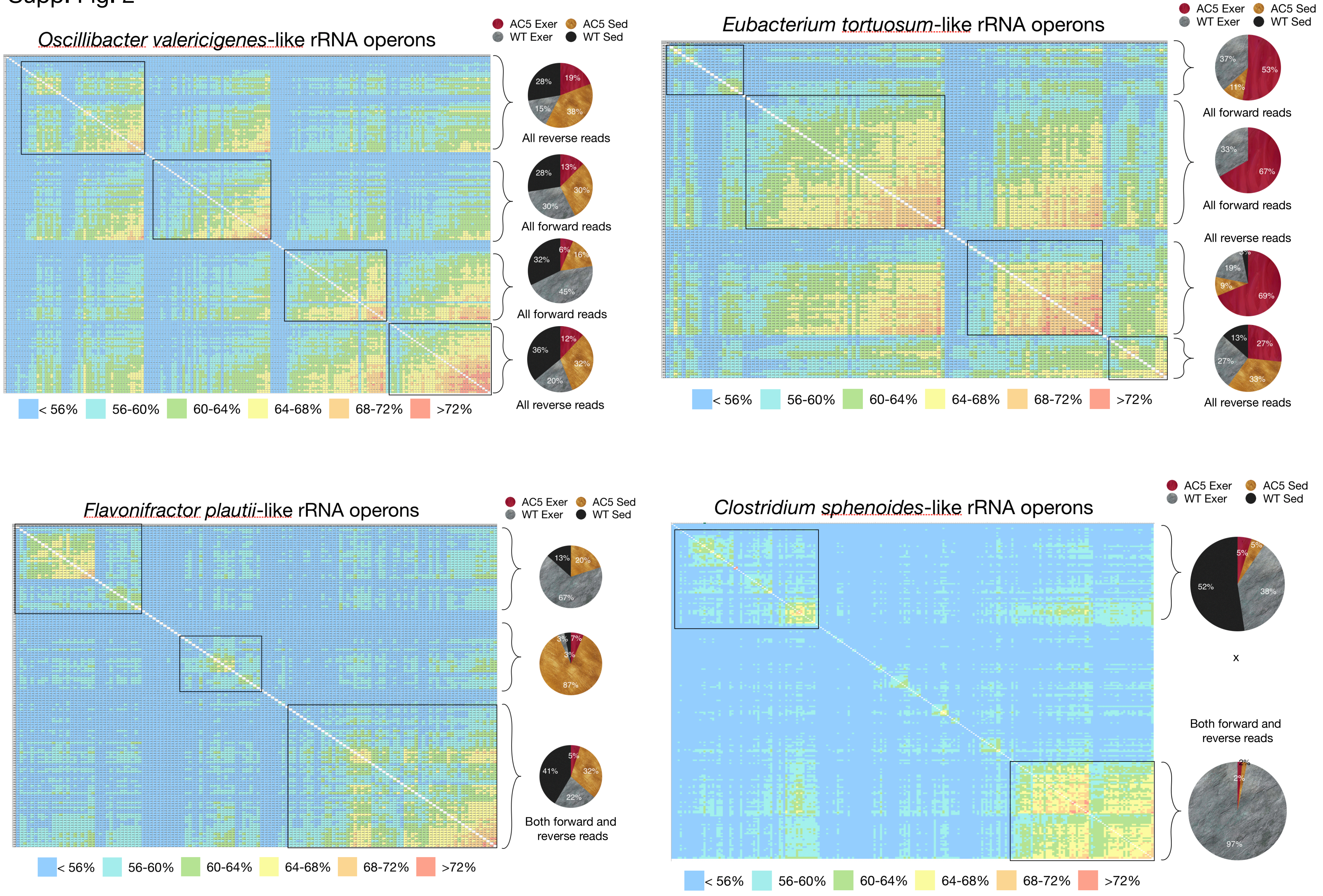

Oscillibacter valericigenes-like rRNA operons
AC5 Exer
AC5 Sed
WT Exer
WT Sed
28%
19%
15%
38%
All reverse reads
28%
13%
30%
30%
All forward reads
32%
6%
16%
45%
All forward reads
36%
12%
32%
20%
All reverse reads
< 56%
56-60%
60-64%
64-68%
68-72%
>72%
Eubacterium tortuosum-like rRNA operons
AC5 Exer
AC5 Sed
WT Exer
WT Sed
37%
53%
11%
All forward reads
33%
67%
All forward reads
All reverse reads
19%
9%
69%
13%
27%
27%
33%
All reverse reads
< 56%
56-60%
60-64%
64-68%
68-72%
>72%
Flavonifractor plautii-like rRNA operons
AC5 Exer
AC5 Sed
WT Exer
WT Sed
13%
20%
67%
3%
7%
3%
87%
41%
5%
32%
22%
Both forward and reverse reads
< 56%
56-60%
60-64%
64-68%
68-72%
>72%
Clostridium sphenoides-like rRNA operons
AC5 Exer
AC5 Sed
WT Exer
WT Sed
5%
5%
52%
38%
x
Both forward and reverse reads
2%
2%
97%
< 56%
56-60%
60-64%
64-68%
68-72%
>72%

Supp. Fig. 3

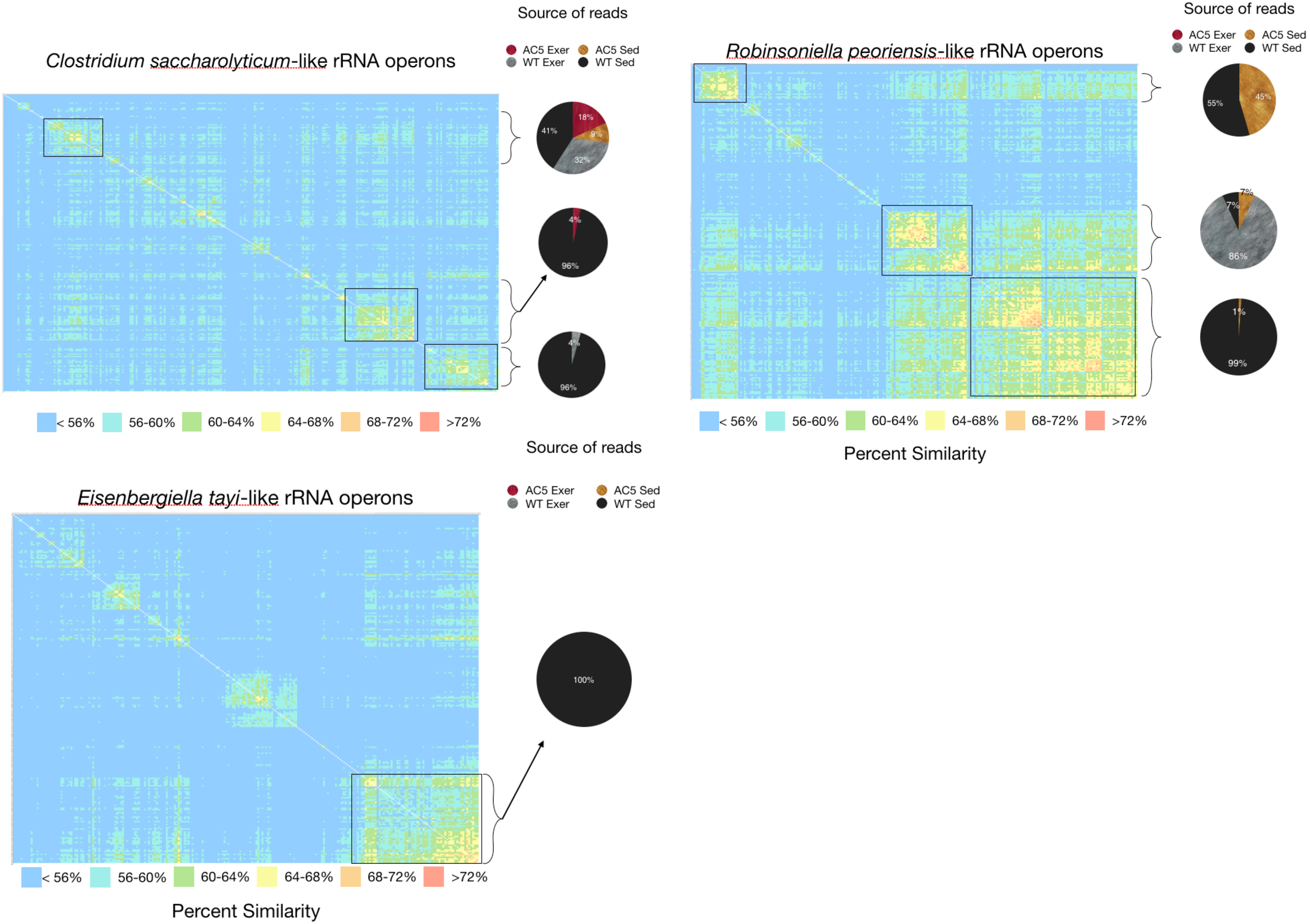

Clostridium saccharolyticum-like rRNA operons
Source of reads
AC5 Exer
AC5 Sed
WT Exer
WT Sed
18%
9%
32%
41%
4%
96%
4%
96%
< 56%
56-60%
60-64%
64-68%
68-72%
>72%
Robinsoniella peoriensis-like rRNA operons
Source of reads
AC5 Exer
AC5 Sed
WT Exer
WT Sed
55%
45%
7%
7%
86%
1%
99%
< 56%
56-60%
60-64%
64-68%
68-72%
>72%
Percent Similarity
Eisenbergiella tayi-like rRNA operons
Source of reads
AC5 Exer
AC5 Sed
WT Exer
WT Sed
100%
< 56%
56-60%
60-64%
64-68%
68-72%
>72%
Percent Similarity

Supp. Fig. 4

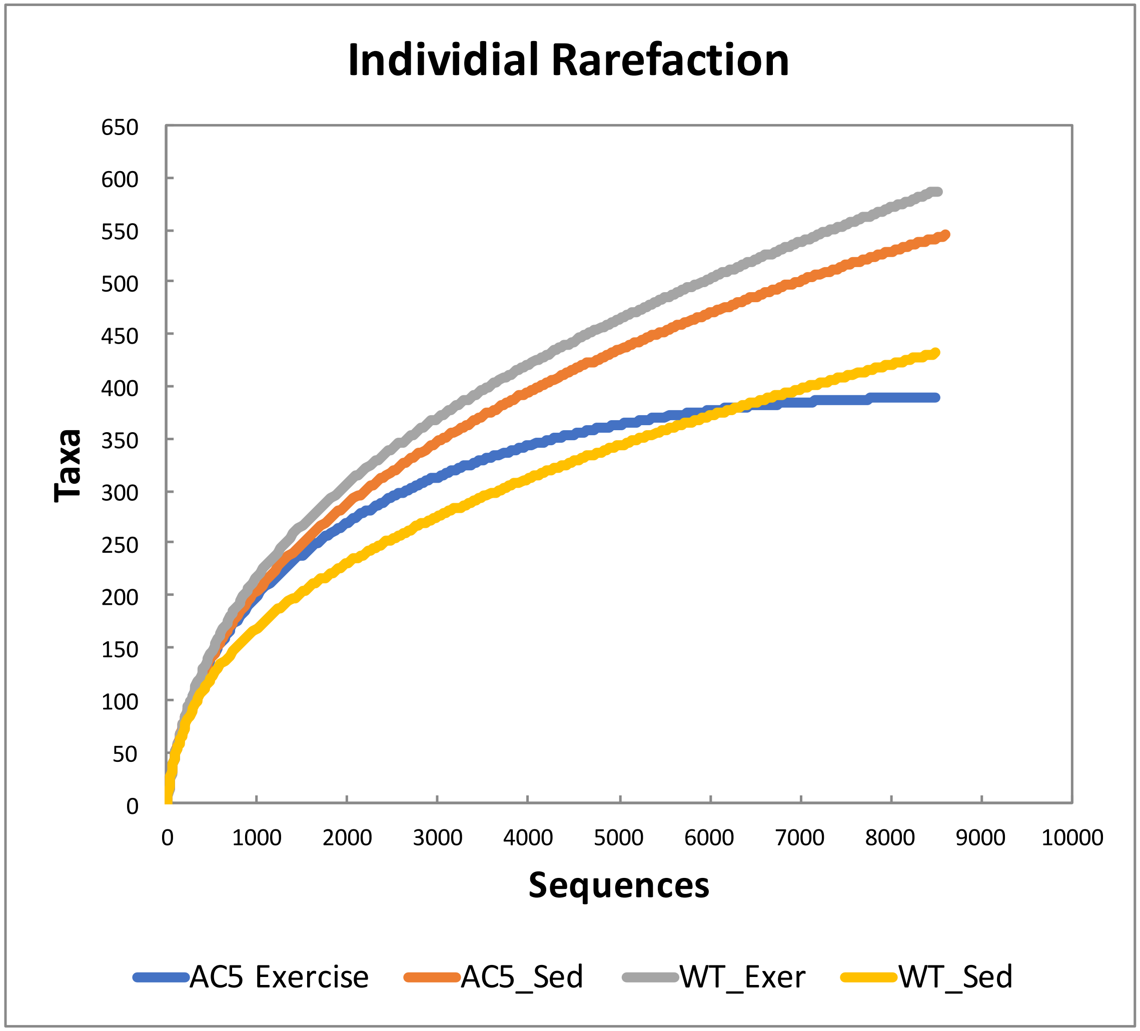

Individial Rarefaction
Taxa
650
600
550
500
450
400
350
300
250
200
150
100
50
0
0
1000
2000
3000
4000
5000
6000
7000
8000
9000
10000
Sequences
AC5 Exercise
AC5_Sed
WT_Exer
WT_Sed

Supp. Fig. 5

A

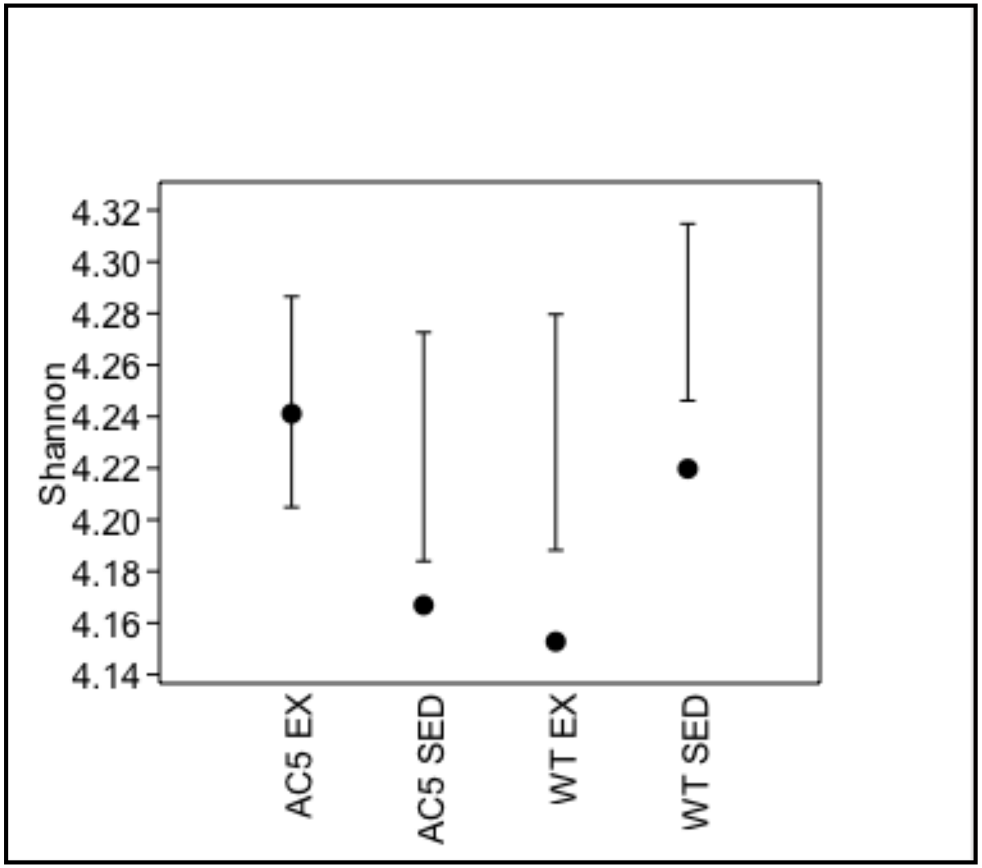

B

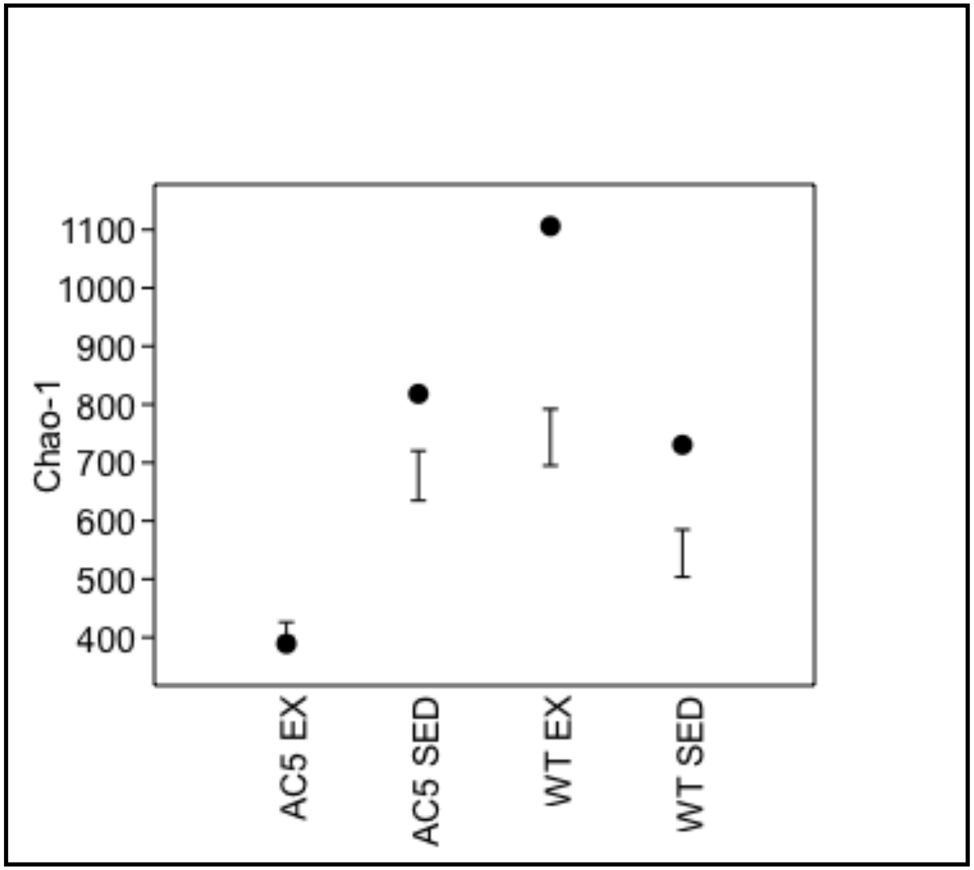

α-Diversity of bacterial communities from feces. (A & B) **A**: Shannon and **B:** Chao1 from normalized read averages from AC5-EX, AC5-SED, WT-EX & WT-SED. Values shown with 95% confidence intervals.

Supp. Fig. 6

Muribaculum species distance matrices

V3-V4 region of16S rRNA gene (275 bp)

	AC5KO Exercise *Muribaculum*	Muribaculum intestinale YL27	Muribaculum intestinale YL27	Muribaculum intestinale YL27	Muribaculum intestinale YL27	WT Sedentary *Muribaculum* sp	WT Sedentary *Muribaculum* sp	AC5KO Sedentary *Muribaculu*	WT Exercise *Muribaculum* sp.	AC5KO Sedentary *Muribaculu*	WT Exercise *Muribaculum* sp.
AC5KO Exercise *Muribaculum sp.* rRNA_A		94	94	94	94	90	90	95	94	93	94
Muribaculum intestinale YL27 rRNA_D	94		100	100	100	93	93	96	97	96	97
Muribaculum intestinale YL27 rRNA_A	94	100		100	100	93	93	96	97	96	97
Muribaculum intestinale YL27 rRNA_B	94	100	100		100	93	93	96	97	96	97
Muribaculum intestinale YL27 rRNA_C	94	100	100	100		93	93	96	97	96	97
WT Sedentary *Muribaculum sp.* rRNA_B	90	93	93	93	93		100	94	94	93	94
WT Sedentary *Muribaculum sp.* rRNA_A	90	93	93	93	93	100		94	94	93	94
AC5KO Sedentary *Muribaculum sp.* rRNA_A	95	96	96	96	96	94	94		96	95	96
WT Exercise *Muribaculum sp.* rRNA_A	94	97	97	97	97	94	94	96		98	100
AC5KO Sedentary *Muribaculum sp.* rRNA_B	93	96	96	96	96	93	93	95	98		98
WT Exercise *Muribaculum sp.* rRNA_B	94	97	97	97	97	94	94	96	100	98	

Near-full length 16S rRNA gene (1330 bp)

	AC5KO Exercise *Muribaculum*	Muribaculum intestinale YL27	Muribaculum intestinale YL27	Muribaculum intestinale YL27	Muribaculum intestinale YL27	WT Sedentary *Muribaculum* sp	WT Sedentary *Muribaculum* sp	AC5KO Sedentary *Muribaculu*	WT Exercise *Muribaculum* sp.	AC5KO Sedentary *Muribaculu*	WT Exercise *Muribaculum* sp.
AC5KO Exercise *Muribaculum sp.* rRNA_A		91	91	91	91	91	91	93	91	91	90
Muribaculum intestinale YL27 rRNA_D	91		100	100	100	92	92	93	94	94	94
Muribaculum intestinale YL27 rRNA_A	91	100		100	100	92	93	93	94	94	94
Muribaculum intestinale YL27 rRNA_B	91	100	100		100	92	93	93	94	94	94
Muribaculum intestinale YL27 rRNA_C	91	100	100	100		92	93	93	94	94	94
WT Sedentary *Muribaculum sp.* rRNA_B	91	92	92	92	92		98	94	93	93	93
WT Sedentary *Muribaculum sp.* rRNA_A	91	92	93	93	93	98		93	93	93	93
AC5KO Sedentary *Muribaculum sp.* rRNA_A	93	93	93	93	93	94	93		94	94	94
WT Exercise *Muribaculum sp.* rRNA_A	91	94	94	94	94	93	93	94		98	98
AC5KO Sedentary *Muribaculum sp.* rRNA_B	91	94	94	94	94	93	93	94	98		98
WT Exercise *Muribaculum sp.* rRNA_B	90	94	94	94	94	93	93	94	98	98	

Parasutterella and *Turicimonas* species distance matrices

V3-V4 region of16S rRNA gene (275 bp)

	T. sp. AC5 Exer_A rRN	T. sp. WT ExerA rRNA	T. muris YL45 rRNA_D	T. muris YL45 rRNA_B	T. muris YL45 rRNA_C	T. muris YL45 rRNA_A	P. sp. AC5KO Exer rRI	P. sp. WT Exer rRNA_	P. sp. AC5KO Sed rRI	P. sp. AC5KO Exer rR	P. sp. WT Exer rRNA_	P. sp. WT Sed rRNA_/	P. sp. WT Sed rRNA_E	P. excrementihominis
T. sp. AC5 Exer_A rRNA A		97	98	98	98	98	90	90	90	90	90	87	87	89
T. sp. WT ExerA rRNA_A	97		98	98	98	98	90	89	91	91	90	88	87	89
T. muris YL45 rRNA_D	98	98		100	100	100	91	91	92	92	92	89	89	91
T. muris YL45 rRNA_B	98	98	100		100	100	91	91	92	92	92	89	89	91
T. muris YL45 rRNA_C	98	98	100	100		100	91	91	92	92	92	89	89	91
T. muris YL45 rRNA_A	98	98	100	100	100		91	91	92	92	92	89	89	91
P. sp. AC5KO Exer rRNA_A	90	90	91	91	91	91		98	97	97	98	92	93	93
P. sp. WT Exer rRNA_A	90	89	91	91	91	91	98		97	96	97	92	92	93
P. sp. AC5KO Sed rRNA_B	90	91	92	92	92	92	97	97		98	98	92	92	94
P. sp. AC5KO Exer rRNA_B	90	91	92	92	92	92	97	96	98		97	92	92	94
P. sp. WT Exer rRNA_B	90	90	92	92	92	92	98	97	98	97		92	92	94
P. sp. WT Sed rRNA_A	87	88	89	89	89	89	92	92	92	92	92		96	98
P. sp. WT Sed rRNA_B	87	87	89	89	89	89	93	92	92	92	92	96		97
P. excrementihominis YIT 11859	89	89	91	91	91	91	93	93	94	94	94	98	97	

Near-full length 16S rRNA gene (1450 bp)

	T. sp. AC5 Exer_A rRN	T. sp. WT ExerA rRNA	T. muris YL45 rRNA_D	T. muris YL45 rRNA_B	T. muris YL45 rRNA_C	T. muris YL45 rRNA_A	P. sp. AC5KO Exer rRI	P. sp. WT Exer rRNA_	P. sp. AC5KO Sed rRI	P. sp. AC5KO Exer rR	P. sp. WT Exer rRNA_	P. sp. WT Sed rRNA_/	P. sp. WT Sed rRNA_E	P. excrementihominis
T. sp. AC5 Exer_A rRNA A		96	97	97	97	97	91	91	91	91	91	91	90	92
T. sp. WT ExerA rRNA_A	96		98	98	98	98	91	91	92	92	92	91	91	92
T. muris YL45 rRNA_D	97	98		100	100	100	92	93	93	93	93	92	92	94
T. muris YL45 rRNA_B	97	98	100		100	100	92	92	93	93	93	92	92	94
T. muris YL45 rRNA_C	97	98	100	100		100	92	93	93	93	93	92	92	94
T. muris YL45 rRNA_A	97	98	100	100	100		92	93	93	93	93	92	92	94
P. sp. AC5KO Exer rRNA_A	91	91	92	92	92	92		98	98	97	98	93	94	95
P. sp. WT Exer rRNA_A	91	91	93	92	93	93	98		98	98	98	94	94	95
P. sp. AC5KO Sed rRNA_B	91	92	93	93	93	93	98	98		98	99	94	94	95
P. sp. AC5KO Exer rRNA_B	91	92	93	93	93	93	97	98	98		98	94	94	95
P. sp. WT Exer rRNA_B	91	92	93	93	93	93	98	98	99	98		94	94	96
P. sp. WT Sed rRNA_A	91	91	92	92	92	92	93	94	94	94	94		97	98
P. sp. WT Sed rRNA_B	90	91	92	92	92	92	94	94	94	94	94	97		98
P. excrementihominis YIT 11859	92	92	94	94	94	94	95	95	95	95	96	98	98	

85%-88% 88%-91% 91%-94% 94%-97% >97%

Percent Similarity

Supplemental Table 1: Summary of MinION run data

Sequencing Run	Total Reads	Basecalled	NCBI Blast	Unclassified
**First**	182,731	52,039	37,346	47,362
**Second**	200,000	34,488	28,360	81,098
**Total**	382,731	86,527	65,706	128,460

Summary data from two MinION sequencing runs for 18 and 22 hours, respectively. Following sequencing, reads were basecalled using Albacore (2.1), sized (between 3.7-5.7 kb) and screened against the NCBI rRNA database using Discontiguous MegaBLAST. Values represents counts of individual reads.
